# Supplementary material for: Histological and histomorphometric aspects of continual intermittent parathyroid hormone administration on osseointegration in osteoporosis rabbit model
Source: PLoS One. 2022 Jun 8;17(6):e0269040. doi: 10.1371/journal.pone.0269040 (PMC9176794; doi:10.1371/journal.pone.0269040)
Supplement: Attachment — Submitted filename: Response to Reviewers20220422.docx [file pone.0269040.s002.docx]

**PONE-D-22-03364**

**Histological and histomorphometric aspect of continual intermittent administration of parathyroid hormone on osseointegration under osteoporosis in rabbit model**

**PLOS ONE**

**Response to Reviewers**

We wish to express our strong appreciation to reviewers’ insightful comments on our manuscript. We feel the comments have helped us significantly improve the present study. We thank for careful reading of our manuscript and constructive criticism. The portions were presented in the revised manuscript with track changes and inserted comments.

**Authors answer**

1. We added the method of anesthesia information in the manuscript with yellow highlights based on [https://journals.plos.org/plosone/s/submission-guidelines#loc-animal-research](https://jpn01.safelinks.protection.outlook.com/?url=https%3A%2F%2Fjournals.plos.org%2Fplosone%2Fs%2Fsubmission-guidelines%23loc-animal-research&data=04%7C01%7Ckazuya17%40hiroshima-u.ac.jp%7C8484ea951d7747010fa608da1d57540c%7Cc40454ddb2634926868d8e12640d3750%7C1%7C0%7C637854558146957713%7CUnknown%7CTWFpbGZsb3d8eyJWIjoiMC4wLjAwMDAiLCJQIjoiV2luMzIiLCJBTiI6Ik1haWwiLCJXVCI6Mn0%3D%7C3000&sdata=c%2BGt%2FBrxYhdJQcnTUhDnvAeIhUeGyJPn2fCu5v08DpI%3D&reserved=0).

2. We have upload our study’s minimal underlying data on figshare site [[10.6084/m9.figshare.19519042](https://jpn01.safelinks.protection.outlook.com/?url=https%3A%2F%2Fdoi.org%2F10.6084%2Fm9.figshare.19519042&data=04%7C01%7Ckazuya17%40hiroshima-u.ac.jp%7C7efe99a0c0ac43275d5508da16f2feb4%7Cc40454ddb2634926868d8e12640d3750%7C1%7C0%7C637847530141935589%7CUnknown%7CTWFpbGZsb3d8eyJWIjoiMC4wLjAwMDAiLCJQIjoiV2luMzIiLCJBTiI6Ik1haWwiLCJXVCI6Mn0%3D%7C3000&sdata=0rsfvwpGIT1Qqy7lbfzFJILOZrest6iHtjow4n%2BhHEE%3D&reserved=0)].

**Review Comments to the Author**

**Reviewer #1:** Dear Authors, congratulations for the article ¨Histological and histomorphometric aspect of continual intermittent administration of parathyroid hormone on osseointegration under osteoporosis in rabbit model¨.

1-1) In my opinion, several sections should be improved so that the study can be better understood. In the abstract, for example, in the first paragraph the objectives of the study should be presented more clearly. There are too many words repeated in the abstract text unnecessarily. Furthermore, no concrete results are presented or described.

**Authors answer 1-1)**

**We revised the abstract chapter with track changes. Also, corrected the graph and included detailed data in the results.**

1-2) The entire article must be proofread for grammar.

**Authors answer 1-2)**

**We had proofreading in English and attached file of the Editing Certificate.**

1-3) The Materials and Methods and Results sections should be better organized and, please, insert the images where they are mentioned in the text.

**Authors answer 1-3)**

**We inserted the figure images in the manuscript.**

1-4) Figure legends should all be rewritten so that they can be better understood by readers.

**Authors answer 1-4)**

**We revised all figure legends (Figure1~9).**

1-5) Using symbols like micro-CT (µCT) is not suitable in many places where it was used and how it was used.

**Authors answer 1-5)**

**We correct the word (we changed from “µCT” to “micro computed tomography; micro-CT”).**

1-6) The discussion chapter is very scattered and confusing, it should be rewritten and better organized.

**Authors answer 1-6)**

**We revised the discussion chapter with track changes.**

1-7) Finally, the authors should present more up-to-date references, several of the references are more than 10 years old.

**Authors answer 1-7)**

**We added recent papers.**

**9. Lane NE. Glucocorticoid-Induced Osteoporosis: New Insights into the Pathophysiology and Treatments. Curr Osteoporos Rep. 2019 Feb;17(1):1-7. doi: 10.1007/s11914-019-00498-x. PMID: 30685820; PMCID: PMC6839409.**

**10. Compston J. Glucocorticoid-induced osteoporosis: an update. Endocrine. 2018 Jul;61(1):7-16. doi: 10.1007/s12020-018-1588-2. Epub 2018 Apr 24. PMID: 29691807; PMCID: PMC5997116.**

**Review Comments to the Author**

**Reviewer #2:**

Ricardo B. V. Fontes, MD, PhD

I had the pleasure of reviewing the submission titled "Histological and histomorphometric aspect of continual intermittent administration of parathyroid hormone on osseointegration under osteoporosis in rabbit model". It is scientifically sound and meets PlosOne publication criteria.

The manuscript needs to be reviewed for English grammar and style.

**Authors answer 2)**

**We had proofreading in English and attached file of the Editing Certificate.**
